# Rapid monitoring of health services use following a policy to switch patients from originator to biosimilar etanercept—a cohort study in British Columbia

**DOI:** 10.1186/s41927-021-00235-x

**Published:** 2022-01-27

**Authors:** Anat Fisher, Jason D. Kim, Greg Carney, Colin Dormuth

**Affiliations:** Department of Anesthesiology, Pharmacology and Therapeutics, University of British Columbia, 2176 Health Sciences Mall, Vancouver, BC V6T 1Z3 Canada

**Keywords:** Arthritis, psoriatic, Arthritis, rheumatoid, Biosimilar pharmaceuticals, Drug switching, Etanercept, Health policy, Insurance coverage, Spondylitis, ankylosing

## Abstract

**Background:**

Drug coverage policies that incentivize switching patients from originator to biosimilar products may result in significant health care savings. Our study aimed to detect early impacts on health services utilization following a mandated switch from originator to biosimilar etanercept in British Columbia (BC), Canada.

**Methods:**

We conducted a prospective, population-based cohort study using linked administrative health data from BC (2010–2020). The policy cohort consisted of patients with inflammatory arthritis who used originator etanercept in 2019, prior to BC’s Biosimilars Initiative switching policy. Three historical cohorts included patients with inflammatory arthritis who used originator etanercept in the years 2016, 2017, and 2018. We compared the daily cumulative incidences of drug refills and outpatient and inpatient services between the policy and historical cohorts. A likelihood ratio sustained (≥ 31 days) at 7.1 or higher compared with the null hypothesis was chosen a priori as a threshold for a potential impact of the policy.

**Results:**

Each cohort contained between 1694 and 1963 patients. We detected several potential impacts: 1) a transient increase in etanercept refills between months three and eight (cumulative incidence difference of + 3.0%); 2) an anticipated increase in visits to physicians of any specialty between months three and eight (+ 2.6%); and 3) an anticipated increase in visits to a rheumatologist from the end of month three onwards (+ 12.8%). The policy had no impact on incidences of switching to a different biologic antirheumatic drug, visits to emergency departments, or admissions to hospitals.

**Conclusions:**

Only transient and/or anticipated increases in drug refills and physician visits were observed during the study period. Additional research on clinical outcomes is recommended to strengthen the evidence that no long-term unintended negative health impacts are associated with BC’s Biosimilars Initiative [switching policy].

**Supplementary Information:**

The online version contains supplementary material available at 10.1186/s41927-021-00235-x.

## Background

Biologic disease-modifying anti-rheumatic drugs (bDMARDs), such as tumor necrosis factor inhibitors, have revolutionized the management of serious health conditions over the last two decades. This therapy has also raised health care costs significantly [1]. Originator etanercept (trade name Enbrel) was the first tumor necrosis factor inhibitor approved to treat patients with rheumatoid arthritis in Canada and the United States. Etanercept is widely prescribed to treat inflammatory arthritis, such as rheumatoid arthritis, psoriatic arthritis, and axial spondyloarthritis [2–4]. Following the recent patent expiration of originator etanercept, the more affordable biosimilar versions of etanercept are available. Switching patients from originator etanercept to its biosimilars as part of routine clinical practice may result in significant public and private drug cost savings [5, 6]. The biosimilar products Brenzys and Erelzi were approved by Health Canada in August 2016 and April 2017 respectively [7]. Both have demonstrated no clinically meaningful differences in quality, efficacy, safety, or immunogenicity compared with originator etanercept in clinical trials [8] or in analyses of real-world data [9]. Treatment persistence with biosimilar versions of etanercept was comparable or longer than with originator etanercept [10, 11]. Even with increasing evidence that switching to biosimilar products is not associated with negative effects on patient health, some patients and physicians have expressed concerns and resisted switching [12–15].

On 27 May 2019 the British Columbia (BC) Ministry of Health implemented Phase 1 of the Biosimilars Initiative, a drug coverage policy that required a non-medical switch from originator etanercept to its biosimilars [16]. To qualify for cost coverage by the provincial drug plan PharmaCare, patients treated with originator etanercept for rheumatoid arthritis, ankylosing spondylitis, or psoriatic arthritis were required to switch to a biosimilar etanercept. During a six-month transition period, BC PharmaCare covered the costs of originator etanercept only for patients with continuous approval of coverage. After the transition period, only the biosimilars were covered, unless a medical exemption was submitted by a physician.

The BC Ministry of Health requested real-time monitoring of the impact of the Biosimilars Initiative on health services utilization. This involved examining the use of health services such as emergency department visits and drugs such as nonsteroidal anti-inflammatory drugs (NSAIDs) or oral steroids, which served as proxies for disease activity in this analysis [17, 18]. Previous studies assessed changes in health services utilization following the launch of a non-medical switching policy for bDMARDs based on estimates from physician surveys [19, 20] or real-world data on etanercept switching [21–24]. These methodologies require significant resources and are limited to a single analysis, which is often delayed. The aim of this rapid monitoring analysis was to detect signals of intended and unintended impacts on health services utilization over a one-year period following the launch of the Biosimilars Initiative for patients treated with originator etanercept [25, 26]. Our study was designed to detect potential signals of unintended negative impacts in an entire population, and not to accept or reject a hypothesis of harm in the presence of a sampling error. If negative impacts were detected, additional analysis was considered. Unintended negative impacts of the policy are, for example, a decrease in refilling etanercept or an increase in switching to other bDMARDs, emergency department visits, or hospitalizations. Intended impacts include an increase in the percentage use of biosimilars in etanercept utilization and an increase in visits to physicians.

## Methods

### Setting

The Canadian province of British Columbia has a universal health system that provides access to medically necessary health services, such as physician and hospital services. In addition, eligible prescription drugs, certain medical supplies, and pharmacy services are covered under the provincial drug plan, PharmaCare. BC residents, regardless of age and income, are eligible to register for PharmaCare coverage. The largest plan, Fair PharmaCare, is income-based, i.e., patients and families are eligible for cost subsidies after reaching their annual deductible level [27].

### Study design and data source

We conducted rapid monitoring of health services utilization [25, 26] using a prospective cohort study design and administrative data from the BC Ministry of Health. The data included population-based, province-wide information on prescriptions filled in all community pharmacies, diagnoses recorded in visits to physicians and other providers, hospital and emergency department records, and medical services registration data. Data were linked using anonymized identifiers. We had no access to data of individuals covered directly by federal health care programs [28] and beneficiaries of the BC First Nations Health Authority Health Benefits Program [29]. Data were analyzed using Oracle SQL Developer and SAS (version 9.4, SAS Institute Inc., Cary, NC, USA) software.

### Study cohorts

We assembled four cohorts of users of the originator etanercept from a source population of individuals enrolled in the provincial health plan. The policy cohort was constructed from all patients who used originator etanercept between 27 November 2018 and 26 May 2019, i.e., during the six months before the launch of the Biosimilars Initiative. Similarly, patients were included in the three historical cohorts if they refilled a prescription for the originator etanercept in the six months before 27 May in the prior years of 2016, 2017, and 2018. Cohort entry occurred on 27 May of each year. We excluded patients who were not targeted by the policy, i.e., patients with psoriasis, those who had discontinued or switched away from originator etanercept, and patients whose drug costs were not covered by PharmaCare. We also excluded patients with short follow-up (< 30 days). For details on exclusion criteria, please refer to Additional file 1: Table S1.

**Table 1:** Identification of historical and policy cohorts: eligible, excluded, and included users of originator etanercept

	Cohort, n (%)			
	Historical			Policy
	2016	2017	2018	2019
Identification period	November 28, 2015 to May 26, 2016^a^	November 27, 2016 to May 26, 2017	November 27, 2017 to May 26, 2018	November 27, 2018 to May 26, 2019
Eligible prescriptions for originator etanercept (patients) during the identification period	11,360 (2423)	11,952 (2476)	11,274 (2266)	10,374 (2065)
Exclusion criteria, number of patients excluded (% of patients identified)^b^				
Diagnosis of psoriasis	112 (4.6)	88 (3.6)	81 (3.6)	69 (3.3)
Low compliance/discontinuers	283 (11.7)	276 (11.1)	245(10.8)	220 (10.7)
Switchers	19 (0.8)	22 (0.9)	10 (0.4)	8 (0.4)
Short follow-up	21 (0.9)	20 (0.8)	16 (0.7)	12 (0.6)
No PharmaCare coverage	110 (4.5)	107 (4.3)	91 (4.0)	62 (3.0)
Final number of etanercept users included in the cohort	1878	1963	1823	1694

^a^2016 was a leap year

^b^For details on exclusion criteria, please refer to Additional file 1: Table S1

### Exposure and outcomes

Patients in the policy cohort were considered exposed to the policy, while patients in the three historical cohorts served as controls. The main outcomes consisted of cumulative incidence, updated daily, of drug and health services use: refills for etanercept (originator or biosimilar); switching to biosimilar etanercept (for the policy cohort only); switching to another bDMARD or a targeted synthetic DMARD (tsDMARD); office visits to a physician (outpatient setting, any specialty); office visits to a rheumatologist (outpatient setting); visits to an emergency department; and hospital admissions. Secondary outcomes included the average cumulative dose of etanercept (originator or biosimilar); and the average cumulative days’ supply for each of the following therapeutic groups: conventional synthetic DMARDs (csDMARDs), NSAIDs, or oral steroids. The full list of drugs is included in Additional file 1: Table S2.

**Table 2:** Demographic characteristics and diagnosis of users of the originator etanercept, by cohort

	Cohort			
	Historical			Policy
Demographic	2016 (n = 1878)	2017 (n = 1963)	2018 (n = 1823)	2019 (n = 1694)
Age median (range), years	59.0 (3.0–94.0)	59.0 (3.0–95.0)	60.0 (3.0–96.0)	61.0 (3.0–97.0)
Female	1181 (62.9)	1232 (62.8)	1126 (61.8)	1017 (60.0)
Diagnosis^a^				
Rheumatoid arthritis	1185 (63.1)	1230 (62.7)	1087 (59.6)	996 (58.8)
Juvenile rheumatoid arthritis	49 (2.6)	63 (3.2)	62 (3.4)	48 (2.8)
Ankylosing spondylitis	188 (10.0)	201 (10.2)	201 (11.0)	181 (10.7)
Psoriatic arthritis	237 (12.6)	265 (13.5)	286 (15.7)	300 (17.7)
Undetermined	219 (11.7)	204 (10.4)	187 (10.3)	169 (10.0)
More than one diagnosis	142 (7.6)	142 (7.2)	130 (7.1)	118 (7.0)

Numbers represent patients (% of cohort) unless otherwise specified

^a^Identified based on at least five visits to a physician or emergency department, or at least one discharge from hospital with a diagnosis code for one of the four conditions in the five years before cohort entry. Rheumatoid arthritis: International Classification of Diseases (ICD)-9 code 714 (except 714.3, 714.4); ICD-10 codes M05, M06 (age at first diagnosis > 18 years). Juvenile rheumatoid arthritis: ICD-9 codes 714, 714.3; ICD-10 codes M05, M06, M08 (age at first diagnosis ≤ 18 years). Ankylosing spondylitis: ICD-9 code 720 (except 720.1–720.9); ICD-10 code M45. Psoriatic arthritis: ICD-9 code 696.0; ICD-10 code M07

### Statistical analysis

For the main outcomes, we computed daily cumulative incidence differences between the policy cohort and the average of the historical cohorts. In the absence of an effect of the Biosimilars Initiative, the cumulative incidence trend for the policy cohort was expected to follow the average cumulative incidence trends for the historical cohorts, i.e., the expected cumulative incidence difference was zero. Since the cumulative incidence differences were measured daily and updated monthly, we used likelihood ratios to assess the “strength” of cumulative incidence differences to facilitate their interpretation as signals versus noise. Unlike test statistics, the interpretation of a likelihood ratio remains the same regardless of how many times the data are updated [30]. The likelihood ratio denoted the likelihood of the observed cumulative incidence difference relative to no difference. For our analysis, a cumulative incidence difference was considered a signal if the likelihood ratio for the observed difference versus no difference was sustained at a threshold of 7.1 (*e*^z=1.96^) or higher for 31 days or longer [25]. This means that the null hypothesis (i.e., cumulative incidence difference of zero) was 7.1-fold less likely than the observed difference. Details of the likelihood ratio calculation are available from the study protocol [25]. Secondary outcomes were descriptive; we plotted cumulative quantity per patient over time of follow-up.

## Results

### Biosimilar uptake in British Columbia

**Fig. 1:**
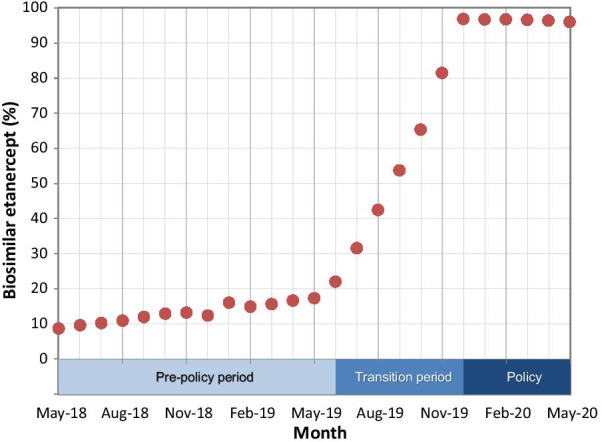
Prescriptions for biosimilar etanercept—percentage of all etanercept prescription refills covered by PharmaCare, by month

During the six-month transition period, we observed a sharp increase in the percentage of prescriptions for the biosimilars among all etanercept prescription refills covered by PharmaCare, from 17.3% (349 of 2016 prescriptions) in May 2019 to 96.9% (1887 of 1948 prescriptions) in December 2019 (Fig. 1).

### Study cohorts

For the rapid monitoring cohorts, we identified between 2065 and 2476 patients with refills for originator etanercept during the six-month identification periods ending on 26 May of each year (Table 1). We excluded between 10.7% and 11.7% (220–283) of the patients in each cohort because they discontinued treatment. After applying additional exclusion criteria, the final historical cohorts included between 1823 and 1963 patients with inflammatory arthritis treated with originator etanercept; the policy cohort contained 1694 patients. Among 2655 patients included in this project, 24% (626) were included in one cohort only, and 40% (1067) were included in all four cohorts. The median age of the patients in each cohort ranged from 59 to 61 years, and most were female (60.0%–62.9%) (Table 2). The most common diagnosis was rheumatoid arthritis (58.8%–63.1%), followed by psoriatic arthritis (12.6%–17.7%).

### Main outcomes

**Fig. 2:**
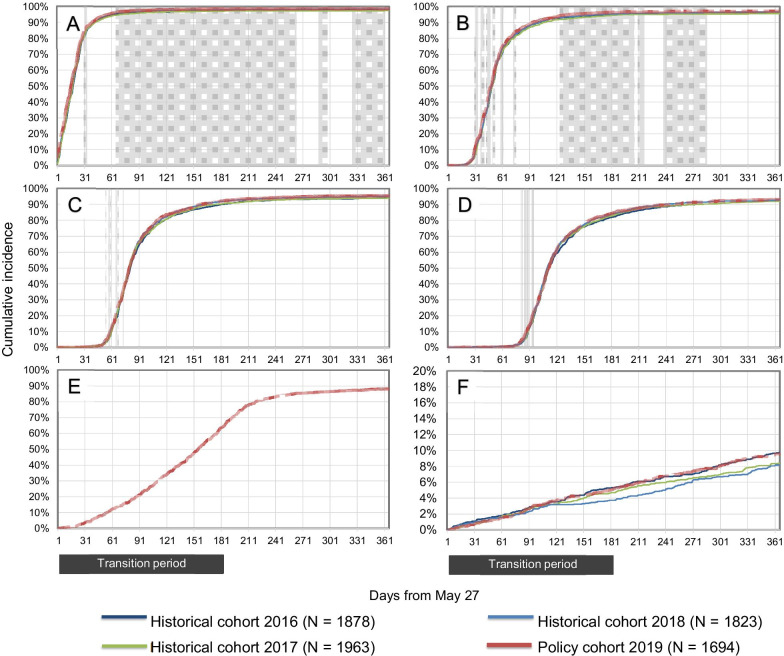
Rapid monitoring of refills of biologic disease-modifying anti-rheumatic drugs following launch of the Biosimilars Initiative. Cumulative incidence of first (**A**), second (**B**), third (**C**) and fourth (**D**) etanercept refills, first refill of an etanercept biosimilar (**E**), and first refill of a different (non-etanercept) biologic, biosimilar, or targeted synthetic disease-modifying anti-rheumatic drug (**F**) during one year of follow-up. Periods with likelihood ratios of 7.1 or higher are shaded. Likelihood ratios were not estimated during the first 31 days of follow-up due to instability in likelihood ratios caused by small numbers

For drug utilizations, we detected a transient increase (up to + 3.0%) in the cumulative incidence difference in the first and second refills of etanercept during follow-up (Fig. 2). The likelihood ratio threshold of > 7.1 was exceeded during days 121 to 261 for the first refill, and day 222 for the second refill. No difference was observed in the cumulative incidence of the third and the fourth refills. By the end of the six-month transition period, 65.1% (1102) of the patients in the policy cohort had switched to a biosimilar version of etanercept, and 88.1% (1493) did so by the end of the one-year follow-up period. Trends in switching to a different bDMARD were similar between the policy and historical cohorts. Between 8.2% and 9.7% (149–183) of the patients in the historical cohorts and 9.6% (162) of the patients in the policy cohort switched to a different bDMARD by the end of the one-year follow-up.

**Fig. 3:**
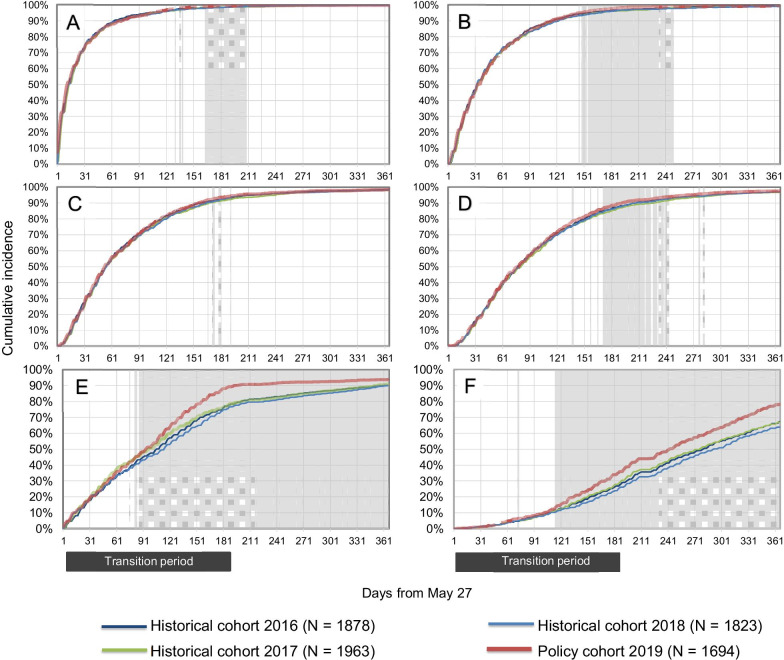
Rapid monitoring of visits to a physician following launch of the Biosimilars Initiative. Cumulative incidence of first (**A**), second (**B**), third (**C**) and fourth (**D**) visits to a physician, and first (**E**) and second (**F**) visits to a rheumatologist during one year of follow-up. Periods with likelihood ratios of 7.1 or higher are shaded. Likelihood ratios were not estimated during the first 31 days of follow-up due to instability in likelihood ratios caused by small numbers

**Fig. 4:**
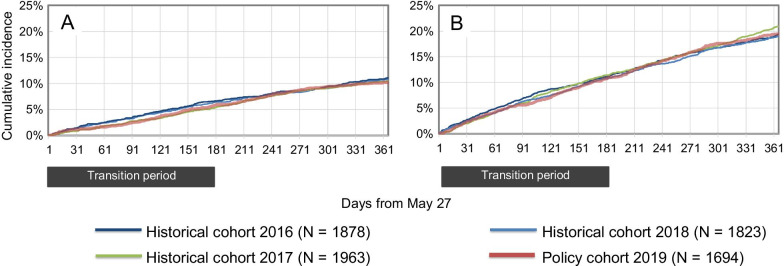
Rapid monitoring of hospital admissions and emergency department visits following launch of the Biosimilars Initiative. Cumulative incidence of admission to hospital (**A**) and visit to an emergency department (**B**) during one year of follow-up. No periods with likelihood ratios of 7.1 or higher were detected

For visits to physicians (Fig. 3), a transient increase of up to + 0.9% was observed in the cumulative incidence difference in first visits to a physician (any specialty, days 168 to 208 of follow-up). A transient increase of up to + 1.7% was detected in second visits (days 148 to 249). No difference was detected in third visits to a physician. Finally, we observed a transient increase of up to + 2.6% in the cumulative incidence difference in fourth visits to a physician (days 171 to 243). Differences in visits to a rheumatologist reached the predefined threshold. The maximal cumulative incidence difference for the first visit to a rheumatologist was + 12.8% (from day 86 onwards). The maximal difference for second visits to a rheumatologist was + 12.7% (from day 111 onwards). No difference was observed in the cumulative incidence of visits to an emergency department or admissions to a hospital between the policy cohort and the three historical cohorts (Fig. 4).

### Secondary outcomes

Patterns of average cumulative quantity of etanercept (originator or biosimilar) dispensed per patient, and average cumulative number of days on csDMARDs, oral steroids, or NSAIDs were similar or lower in the policy cohort compared with the historical cohorts (Additional file 1: Figs. S1–S4).

## Discussion

In this prospective study, we observed an intended increase in the use of biosimilars of etanercept following the launch of the Biosimilars Initiative as most users of originator etanercept switched to the biosimilar products during the six-month transition period. In the monitoring of health services utilization using a pre-defined likelihood ratio threshold of 7.1, we observed anticipated changes, such as an increase in visits to a physician (any specialty) and to a rheumatologist [19, 20, 22, 31]. Other changes were either minor and transient, i.e., a transient increase in etanercept refills, or the direction of the change in utilization suggested a possible benefit. Specifically, patients in the policy cohort had fewer days on csDMARDs, oral steroids, and NSAIDs, which may suggest better control of disease severity [17, 18].

This study has several strengths. First, we were able to monitor the policy and detect change in health services utilization as soon as data were available. We provided policymakers with monthly reports, each with only a one-month lag time. Using likelihood ratios instead of test statistics allowed us to update the analyses and compare utilization daily without having to correct for repeated measures and prior knowledge. A particular strength of this study is the use of a province-wide, population-based dataset that includes data collected systematically and prospectively. Inherent limitations of this study are the absence of a control for possible differences between cohorts, as well as possible correlation between observations of patients included in more than one cohort. Controlling for covariance between cohorts could have caused the likelihood ratios to increase, and cumulative incidence differences with likelihood ratios less than 7.1 would have triggered a signal. The use of administrative data for research also has limitations, including inaccuracies in diagnosis or adherence to drugs, and the absence of information on predictors such as disease severity or smoking.

Several other studies examined the effect of a non-medical switch from originator etanercept to a biosimilar, many of which focused on treatment discontinuation [9, 10, 21, 23, 24, 32–38]. A single study from Denmark examined the change in health services utilization after implementation of a mandatory non-medical switching policy for etanercept [22]. The Danish study found that the use and costs of outpatient services increased after switching patients from originator etanercept to a biosimilar, whereas costs of admissions and drugs decreased. Increases in visits to a physician were also observed in infliximab studies from Italy and Turkey [31, 39]. A cohort study from Italy found an 83% increase in visits to specialists, especially rheumatologists, in patients who switched to the biosimilar infliximab [31]. In a crude analysis from Turkey, health services costs were significantly higher in outpatient settings (*P* = 0.005) for patients who switched to biosimilar infliximab compared with those who continued with originator infliximab [39]. In our study, we found that switching from the originator etanercept to biosimilar products was associated with a transient increase in visits to a physician of any specialty, which diminished within a few weeks of the end of the transition period. It is possible that this increase in physician visits was in patients seeking to discuss treatment options before the switch. An increase in visits after switching, on the other hand, may have indicated unintended negative impacts of a policy. Further research with a design similar to the Danish studies [22, 40, 41] could provide a better understanding of this change in British Columbia.

## Conclusions

Using rapid monitoring of drug and health services utilization, we were able to provide policymakers in British Columbia with real-time data on the impact of the Biosimilars Initiative for users of etanercept. We did not find permanent unintended changes in health services utilization, which suggests that switching to the biosimilar etanercept had minimal impacts on patient health. Additional research on clinical outcomes is recommended to strengthen the evidence that no long-term unintended negative health impacts are associated with switching from originator etanercept to its biosimilars.

## Supplementary Information

**Additional file 1.** **Supplementary Table 1.** Cohorts construction: exclusion criteria. **Supplementary Table 2.** Drugs by therapeutic group. **Supplementary Figure 1.** Cumulative quantity of etanercept (originator or biosimilar) dispensed (mg per patient) over the follow-up period, by cohort. **Supplementary Figure 2.** Cumulative number of days on conventional synthetic disease modifying anti-rheumatic drugs (csDMARDs) per patient, over the follow-up period, by cohort. **Supplementary Figure 3.** Cumulative number of days on oral steroids per patient, over the follow-up period, by cohort. **Supplementary Figure 4.** Cumulative number of days on nonsteroidal anti-inflammatory drugs (NSAIDs) per patient, over the follow-up period, by cohort.

## Data Availability

The authors do not have permission to share data from this study. The data that support the findings of this study are available from PopData BC (https://www.popdata.bc.ca/), but restrictions apply to the availability of these data, which were used under licence for the current study, and so are not publicly available.

## Abbreviations

BC: British Columbia
bDMARD: Biologic disease-modifying anti-rheumatic drug
csDMARD: Conventional synthetic disease-modifying anti-rheumatic drug
NSAIDs: Nonsteroidal anti-inflammatory drugs
tsDMARD: Targeted synthetic disease-modifying anti-rheumatic drug

## Declarations

### Standards of reporting

The study is compliant with the STROBE standard of reporting checklist for observational studies and the SAMPL Guidelines for reporting of basic statistical analyses.
