# Supplementary material for: Rapid monitoring of health services use following a policy to switch patients from originator to biosimilar etanercept—a cohort study in British Columbia
Source: BMC Rheumatol. 2022 Jan 27;6:5. doi: 10.1186/s41927-021-00235-x (PMC8793256; doi:10.1186/s41927-021-00235-x)

**Rapid Monitoring of Health Services Use Following a Policy to Switch Patients from Originator to Biosimilar Etanercept – A Cohort Study in British Columbia**

Supplementary Material

**Supplementary Table 1.** Cohorts construction: exclusion criteria

| **Exclusion criteria** | **Details** |
| --- | --- |
| Psoriasis (unless they had psoriatic arthritis) | Psoriasis   - At least 1 proxy for psoriasis in the 5 years before and including the originator refill date:   - Discharge from hospital or emergency department with a diagnosis of psoriasis (ICD-10 code L40);   - At least 2 physician visits with a diagnosis of psoriasis, within 1 year (ICD-9 code 696);   - At least 5 visits to a dermatologist. - At least 1 originator etanercept refill prescribed by a dermatologist during the identification period.   Psoriatic arthritis   - At least 1 proxy for psoriatic arthritis in the 5 years before and including the originator refill date:   - Discharge from hospital or emergency department with a diagnosis of psoriatic arthritis (ICD-10 codes M07, L40.5);   - At least 2 physician visits with a diagnosis of psoriatic arthritis (ICD-9 code 696.0) within 1 year;   - At least 2 visits to a rheumatologist. - At least 1 originator etanercept refill prescribed by a rheumatologist during the identification period |
| Discontinued the originator etanercept before cohort entry | Without remaining supply of the originator etanercept on 27 May, or without a refill in the 56 days (twice the median days’ supply) before 27 May (from 1 April to 26 May). |
| Switched to another biologic or targeted synthetic disease-modifying antirheumatic drug before cohort entry | Dispensing of any of the following drugs between the originator refill date and 26 May: adalimumab, infliximab, certolizumab, golimumab, abatacept, tocilizumab, anakinra, tofacitinib, rituximab, ustekinumab, secukinumab, ixekizumab, brodalumab, or guselkumab. |
| Switched to a biosimilar etanercept before cohort entry | Dispensing of an etanercept biosimilar (Brenzys^®^ or Erelzi^®^) between the originator refill date and 26 May. |
| Short follow-up | End of health plan enrollment during the first month of follow-up (from 28 May to 27 June). |
| No PharmaCare coverage | Zero originator etanercept refills accepted by PharmaCare during the identification period. |

**Supplementary Table 2.** Drugs by therapeutic group

| **Therapeutic group** | **Generic drugs** |
| --- | --- |
| Biologic and targeted synthetic disease-modifying antirheumatic drugs (bDMARD and tsDMARD) | Adalimumab, infliximab, certolizumab, golimumab, abatacept, tocilizumab, anakinra, tofacitinib, rituximab, ustekinumab, secukinumab, ixekizumab, brodalumab, and guselkumab |
| Conventional synthetic disease-modifying antirheumatic drugs (csDMARDs) | Methotrexate, hydroxychloroquine, leflunomide, sulfasalazine, minocycline, azathioprine, auranofin, chloroquine, cyclophosphamide, cyclosporine, gold sodium thiomalate, mycophenolate, and penicillamine |
| Oral steroids | Cortisone, dexamethasone, hydrocortisone, betamethasone, fludrocortisone, methylprednisolone, prednisone, prednisolone, and triamcinolone |
| Nonsteroidal anti-inflammatory drugs (NSAID) | Aspirin, celecoxib, diclofenac, diflunisal, etodolac, ibuprofen, indomethacin, ketoprofen, nabumetone, naproxen, oxaprozin, piroxicam, salsalate, sulindac, and tolmetin |

**Supplementary Figure 1.** Cumulative quantity of etanercept (originator or biosimilar) dispensed (mg per patient) over the follow-up period, by cohort


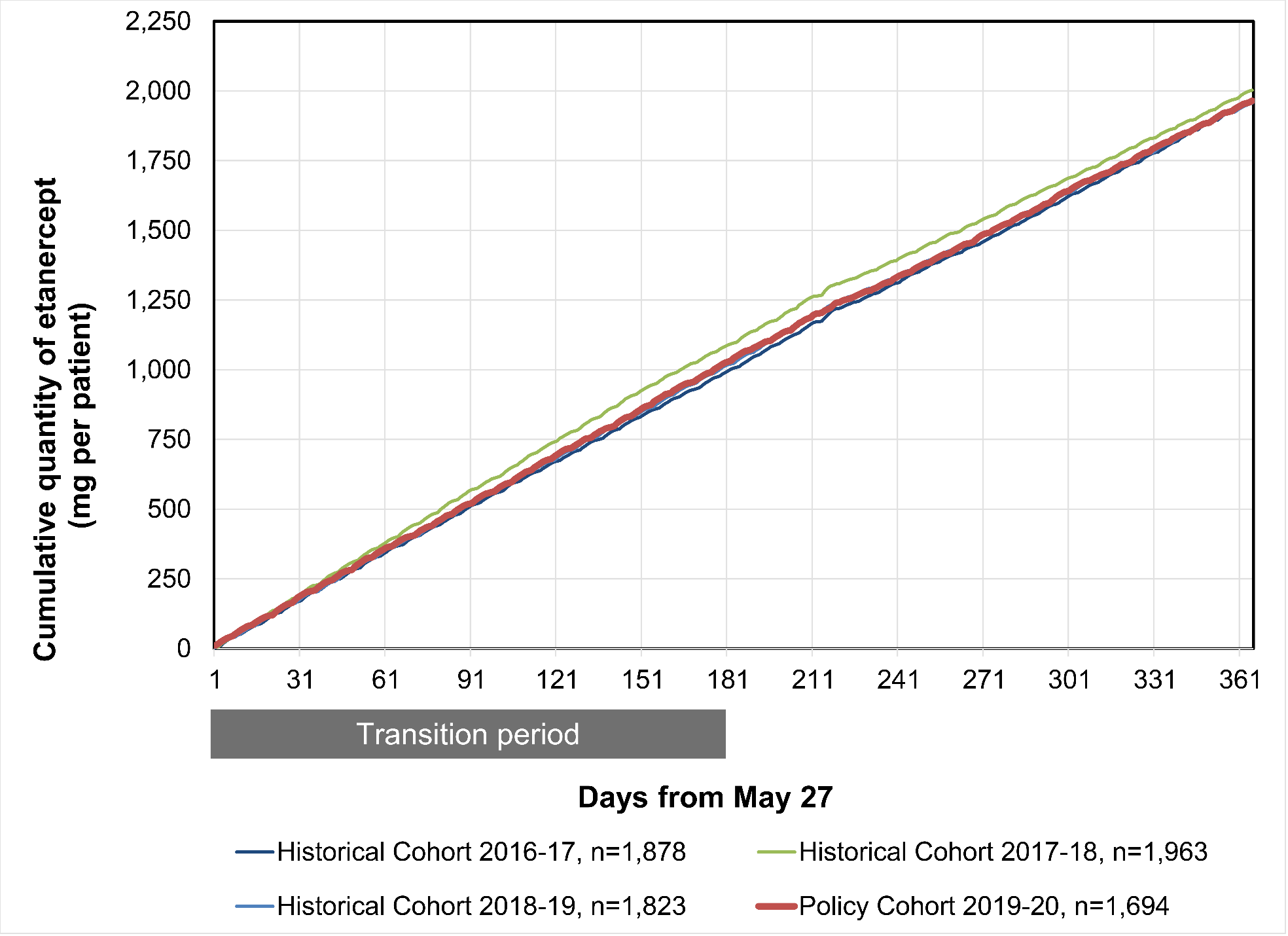


**Supplementary Figure 2.** Cumulative number of days on conventional synthetic disease modifying anti-rheumatic drugs (csDMARDs) per patient, over the follow-up period, by cohort


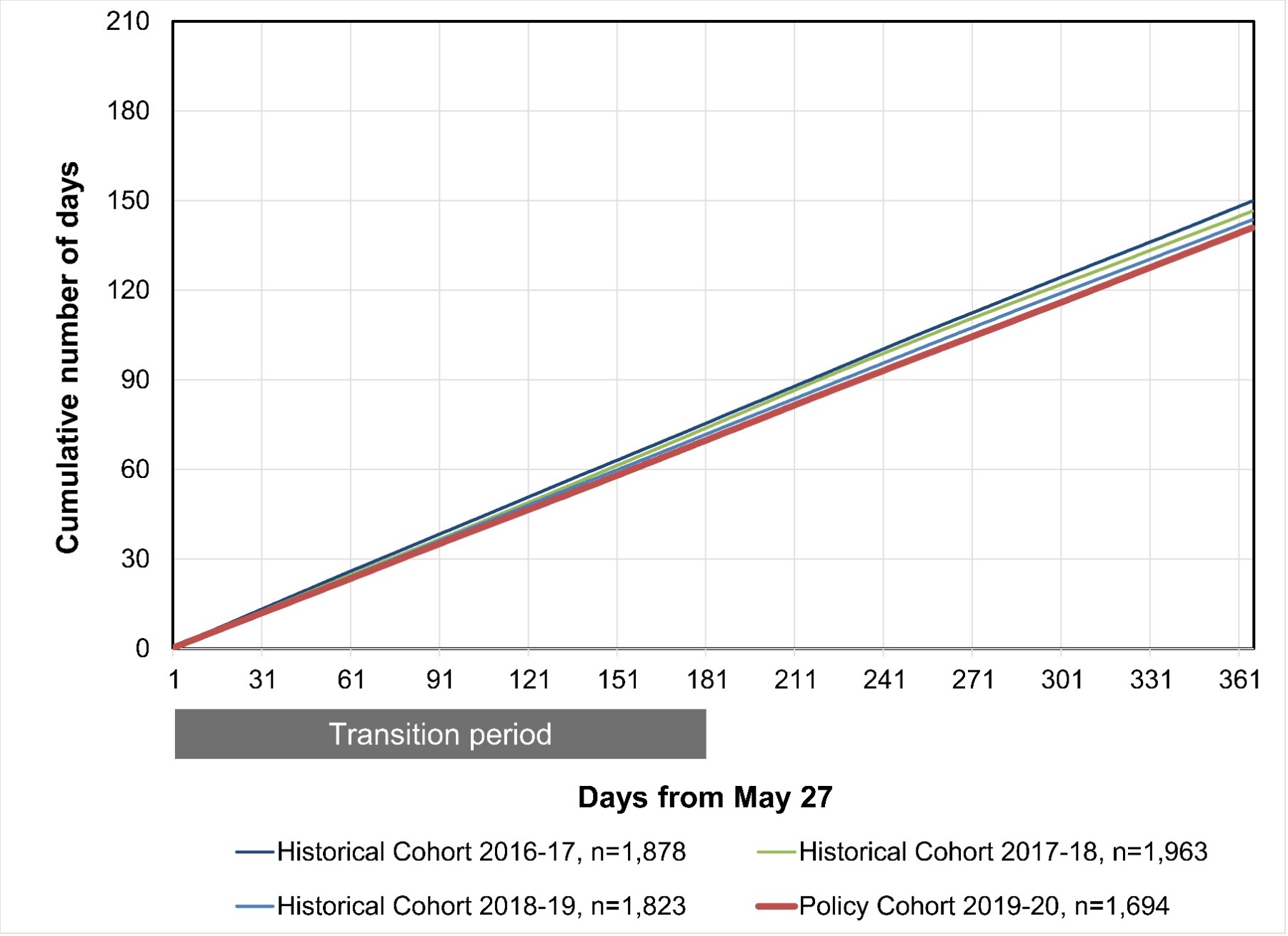


**Supplementary Figure 3.** Cumulative number of days on oral steroids per patient, over the follow-up period, by cohort


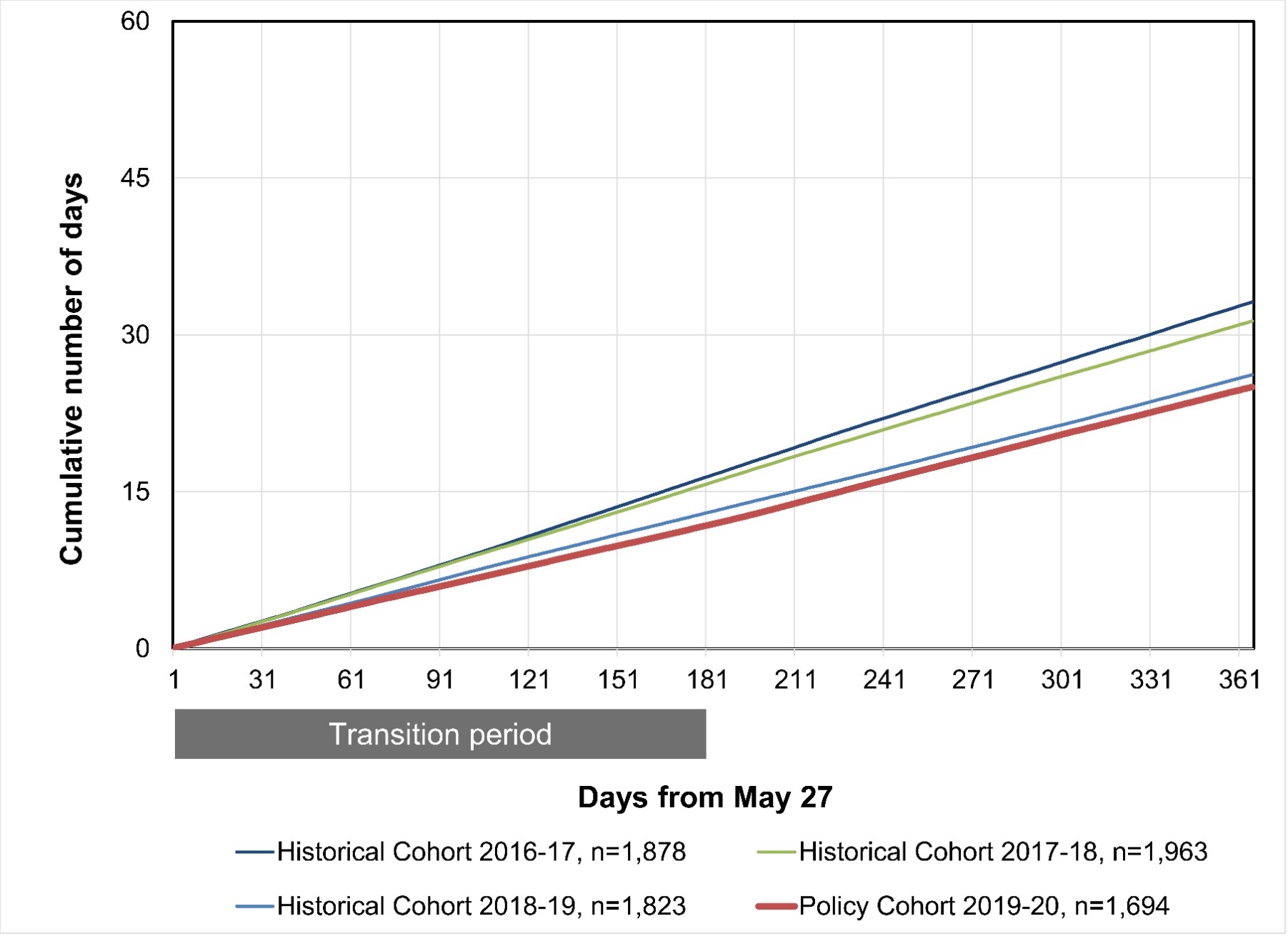


**Supplementary Figure 4.** Cumulative number of days on nonsteroidal anti-inflammatory drugs (NSAIDs) per patient, over the follow-up period, by cohort


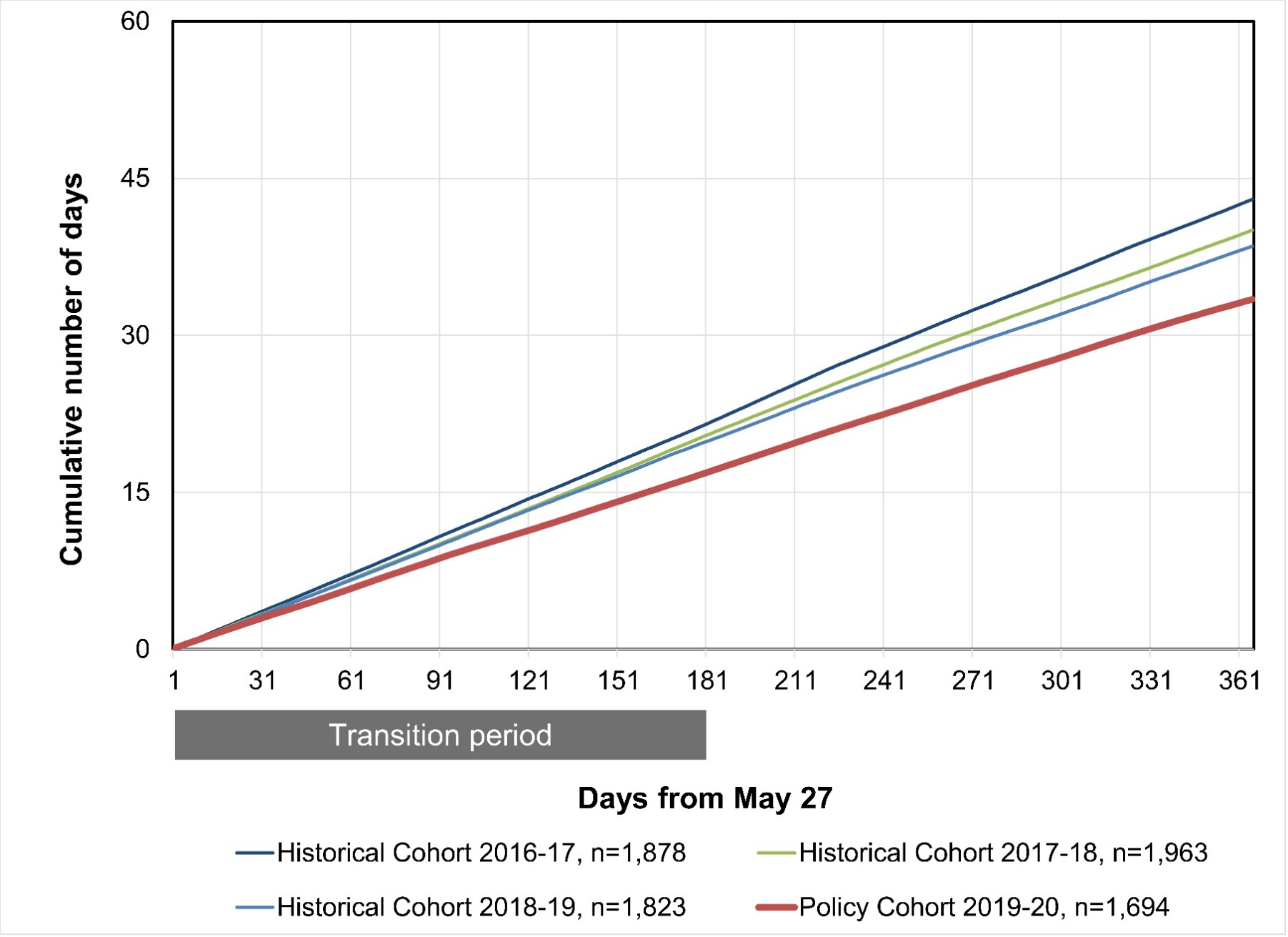

Supplement: Supplementary file 1 — Additional file 1. Supplementary Table 1. Cohorts construction: exclusion criteria. Supplementary Table 2. Drugs by therapeutic group. Supplementary Figure 1. Cumulative quantity of etanercept (originator or biosimilar) dispensed (mg per patient) over the follow-up period, by cohort. Supplementary Figure 2. Cumulative number of days on conventional synthetic disease modifying anti-rheumatic drugs (csDMARDs) per patient, over the follow-up period, by cohort. Supplementary Figure 3. Cumulative number of days on oral steroids per patient, over the follow-up period, by cohort. Supplementary Figure 4. Cumulative number of days on nonsteroidal anti-inflammatory drugs (NSAIDs) per patient, over the follow-up period, by cohort. [file 41927_2021_235_MOESM1_ESM.docx]
